# Effect of Thermal Treatment on the Self-Assembly of Wheat Gluten Polypeptide

**DOI:** 10.3390/molecules28020834

**Published:** 2023-01-14

**Authors:** Hao Liu, Jingxuan Wang, Mei Liu, Xia Zhang, Ying Liang, Jinshui Wang

**Affiliations:** 1 College of Biological Engineering, Henan University of Technology, Zhengzhou 450001, China; 2 College of Food Science and Engineering, Henan University of Technology, Zhengzhou 450001, China

**Keywords:** self-assembly, wheat gluten polypeptide, thermal treatment

## Abstract

Self-assembled fibrillation of wheat gluten is a common phenomenon in the daily production and processing of wheat flour products. The driving forces for its formation and the factors that influence the morphology of fibrils have not been thoroughly investigated. In this study, the effect of three bonding changes (breaking hydrogen bonds, strengthening hydrophobic interactions, and SH-SS exchange reactions) on gluten polypeptide (GP) fibrillation was simulated by adjusting the heating temperature (room temperature (RT), 45 °C, 65 °C, and 95 °C). The results showed that the breakage of hydrogen bonds could induce conformational transitions in GPs and help to excite fibrillation in GPs. Strengthened hydrophobic interactions significantly contributed to the fibrillation of GPs. Covalent crosslinks generated by SH-SS exchange reactions might also promote the fibrillation of GPs. GPs with different degrees of hydrolysis (4.0%, 6.0%, and 10.0%, represented by DH 4, DH 6, and DH 10, respectively) presented different extents of fibrillation, with DH 10 GPs having a higher propensity to fibrillation than DH 4 and DH 6 GPs. The results of Fourier’s transform infrared spectroscopy indicated that hydrophobic interactions drive the transition from a random coil and α-helix to a β-sheet. In addition, hydrophobic interactions also drive the intermolecular polymerization of GPs, resulting in larger molecular weight aggregates. The morphology presented by transmission electron microscopy showed that the greater the DH, the stronger the tendency for the worm-like aggregation of GPs.

## 1. Introduction

Self-assembly is the spontaneous formation of ordered structures of basic structural units (molecules, nanomaterials, substances on the micron, or larger scale) without human intervention [1]. In the process of self-assembly, the basic structural units are spontaneously organized or aggregated into a stable structure with a regular geometrical appearance, based on non-covalent or weak covalent bonding interactions [2]. Self-assembly processes are ubiquitous in nature. Involved in the crystal formation, cell growth, and the synthesis of nanomaterials [3,4,5]. It shows the great potential for application in biology, materials, medicine, and food.

Amyloid fibers (AFs) are mainly formed by short polypeptides or protein molecules through a highly ordered self-assembly, which is known for its rich β-folding and high thermodynamic stability [6]. Under the appropriate environment, almost all proteins can form AFs. However, the conditions under which different proteins form AFs are different, as are the morphological and functional properties of the AFs they form [7]. Jansens et al. investigated the behavior of amyloid aggregation in egg white proteins. They found that the presence of disulfide bonds under heating facilitated the formation of amyloid aggregates with a nucleus-like structure surrounded by loose protein fragments [8]. For plant proteins, wheat gluten contains high levels of glutamine (with a self-assembling propensity) and native β-sheets that facilitate the AF formation [9,10]. However, the complex structure and water insolubility of wheat gluten inhibit the formation, extraction, and analysis of AFs. Xu et al. found that modified soy protein-derived AFs could form well-structured hydrogels with a homogeneous network and that the complexes were non-toxic and antibacterial [11]. Unlike pathological AFs, food-derived AFs have a high stiffness and multiple active functional groups and therefore have promising applications in food emulsification, thickening, foaming, and gelling [12]. As a bulk cereal by-product, the study of the amyloid aggregation behavior of gluten has a practical significance and economic benefit.

Thermal treatment is an ordinary operation in the food processing of wheat-based products. As an essential ingredient in wheat-based products, the changes in gluten during thermal processing have a significant impact on the sensory, structural, and functional properties of the product [13,14]. The changes in molecular weight, aggregation properties, and functional characteristics of gluten proteins arise from changes in the secondary, tertiary, and quaternary structure caused by intra- and intermolecular non-covalent (hydrophobic interactions, hydrogen bonds, van der Waals, and electrostatic interactions) and covalent bonds (coordination and disulfide bonds) recombination during thermal treatment [15]. When heated above 40 °C, the solubility of gluten in water is reduced, owing to the disruption of hydrogen bonds, causing protein unfolding and the exposure of hydrophobic groups [16]. The polymerization of glutenin is driven by hydrophobic interactions when temperatures reach 60 °C [17]. When heating exceeds 90 °C, the free SH groups embedded in the gluten are further exposed, triggering the covalent cross-linking between glutenin and gliadin and thus promoting the polymerization of gluten [18,19]. It is evident that different temperatures trigger different bond changes, and that bond changes are effective drivers of variations in the properties, structure, and function of gluten proteins. However, the effect of bonds (covalent and non-covalent bonds) on the formation of AFs by gluten self-assembly has not been reported.

Wheat gluten has a complex spatial structure and is poorly soluble in water. Moreover, the polymerization of gluten after heating makes it more challenging to extract the AFs. Here, we used gluten-hydrolyzed polypeptides as the subject of this study. By controlling the heating temperature, different interaction forces were simulated to clarify their influence on forming AFs via peptide self-assembly.

## 2. Results and Discussion

### 2.1. Effect of the Different Thermal Effects on the Formation of AFs from GPs

**Figure 1:**
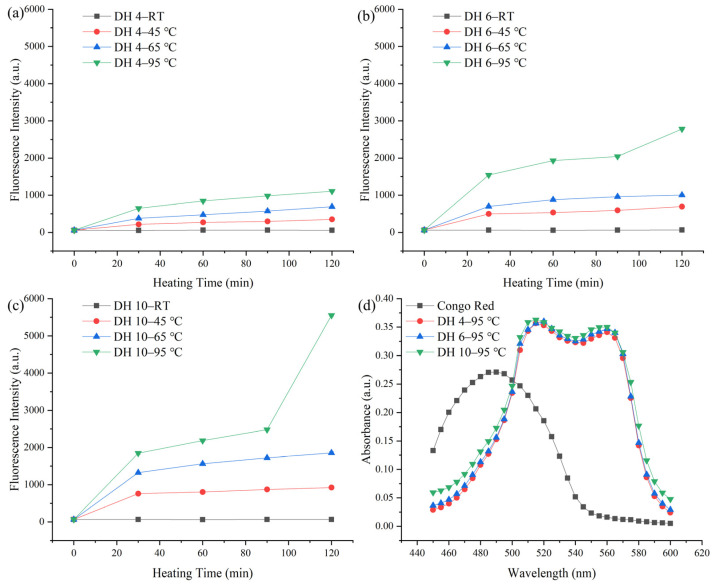
ThT fluorescence intensity of the degree of hydrolysis (DH) 4.0% (**a**), DH 6.0% (**b**), and DH 10.0% (**c**) GPs during heating. Congo red spectrum (**d**). RT, 45, 65, and 95 represent the room temperature, 45 °C, 65 °C, and 95 °C, respectively.

ThT can bind specifically to the β-sheet in AFs, and the amount of binding is proportional to the amount of AFs [20]. The content of the AFs formed by the self-assembly of GPs was quantified using ThT dye fluorescence, as shown in Figure 1.

The fluorescence intensity increased significantly with heating, indicating that heating induced the formation of AFs from GPs. As shown in Figure 1a, the amount and rate of construction of AFs varied by temperature. A significant increase in the fluorescence intensity was observed when heated at 45 °C, compared to room temperature (RT, Figure 1a). This indicated that a disruption of the hydrogen bonds contributed to the formation of AFs. When the temperature reached 45 °C, the hydrogen bonds were broken, leading to the unfolding of the protein structure, which facilitated the structural reorganization of the protein [21]. Above 45 °C, the increase in temperature would lead to an increase in the hydrophobic interactions [22]. The higher the temperature, the faster the rate of AF synthesis (65 °C and 95 °C, Figure 1a), indicating that the increased hydrophobic interactions contribute to the formation of AFs.

When the temperature did not exceed 65 °C, DH 6 GPs and DH 10 GPs (Figure 1b,c) exhibited similar patterns of variation to DH 4 GPs (Figure 1a). GPs with a greater DH (DH 6 and 10) had a higher fluorescence intensity than the GPs with a lower DH (DH 2). This is consistent with Lambrecht et al., who found that DH 6 wheat gluten-derived peptides had a higher propensity for fibrillation than DH 4 wheat gluten-derived peptides [23]. Smaller molecular weight peptides were more susceptible to driving forces, which might explain their greater fibrillation propensity. In addition, a CR spectral shift assay is widely used for the qualitative and quantitative analyses of AFs [12]. The spectra of CR hybrid solutions of DH 4, DH 6, and DH 10 GPs heated at 95 °C for 120 min were measured, as shown in Figure 1d. Following the mixing with CR, DH 4, DH 6, and DH 10 exhibited similar spectra, with variations in the peak height and positions, compared to the original CR spectra. The peak shifted from its intrinsic peak position of about 480 nm to about 510 nm, and a second peak appeared between 560 and 580 nm. This is consistent with the results of Klunk et al., indicating the formation of an ordered β-sheet structure [24]. In terms of the peak height, the DH 10 has a slightly higher peak height, and there was no significant difference between the DH 4 and DH 6 peak heights. This indicated that all GPs with a different DH underwent fibrillation transformation after heating at 95 °C for 120 min [9]. Still, the degree of transformation was probably higher in DH 10 than in DH 4 and DH 6.

### 2.2. Effect of Different Thermal Effects on the Secondary Structure of GPs

FT-IR spectroscopy by studying the amide I region (1600–1700 cm^−1^) is a common method for characterizing the secondary structure of the protein. According to Ridgley et al., 1605–1625 cm^−1^ were a high strand density β-sheet (usually indicated fibrillation), 1625–1635 cm^−1^ were a low strand density β-sheet (usually indicated native or non-amyloid β-structure), 1635–1644 cm^−1^ were the random coil, 1645–1665 cm^−1^ were an α-helix, and 1666–1699 cm^−1^ were a β-turn or antiparallel β-sheet [25]. The FT-IR spectroscopy of GPs was measured to reveal the effect of thermal treatment on the conformation of AFs, as shown in Table 1 and Figure S1 (Supplementary Materials).

Thermal treatment had a significant (*p* < 0.05) effect on the secondary structure of GPs. Compared to the GPs left at RT for 120 min, heating induced a β-sheet conformational shift, with a natural transformation to a dense β-sheet structure (fibrillation). The natural β-sheets in wheat protein contributed to its self-assembled fibrillation [9], which may account for the shift in the β-sheet from natural to fibrillated upon heating the GPs. With the increasing temperature from 45 °C to 95 °C, the content of the β-sheets (fibrillation) in DH 4 GPs significantly increased (*p* < 0.05) from 12.19% to 13.61%. In contrast, the content of the random coil and α-helix decreased (*p* < 0.05) from 19.12% to 18.46% and from 29.28% to 26.42%, respectively. Consistent with the findings of Bhattacharya et al., in their study of the self-assembled fibrillation of ovalbumin, it was found that heating ovalbumin reduced the number of α-helices and random coils and increased the number of β-sheets [26]. This suggested that the transition of α-helices and random coils into β-sheets may have occurred during the fibrillation process [27]. The β-sheets content of DH 4, DH 6, and DH 10 GPs did not change significantly when the temperature was below 45 °C. Furthermore, no correlation was observed between the DH (in DH-6 and DH-10) and the β-sheets content at a particular temperature (65 °C). However, a correlation was observed at 95 °C, where the β-sheets content increased significantly with the increasing DH. This suggested that the secondary structure of GPs was influenced, not only by the temperature, but also by the DH, with the effect of the DH being more pronounced at higher temperatures. In addition, the content of the random coils and α-helices in DH 10 dramatically reduced from 17.52% to 15.55% and 26.67% to 23.21%, when temperature increased from 65 °C to 95 °C, while those in DH 4 and DH 6 slightly reduced. It is reasonable to infer that breaking the hydrogen bond (by heating at 45 °C) only promoted the ordered aggregation of the original β-sheets. In contrast, enhanced hydrophobic interactions changed the conformation of the GPs to form more ordered β-sheet structures, and this effect was more pronounced, the larger the DH of the GPs.

### 2.3. Effect of the Different Thermal Effects on the Molecular Weight Distribution of GPs

**Figure 2:**
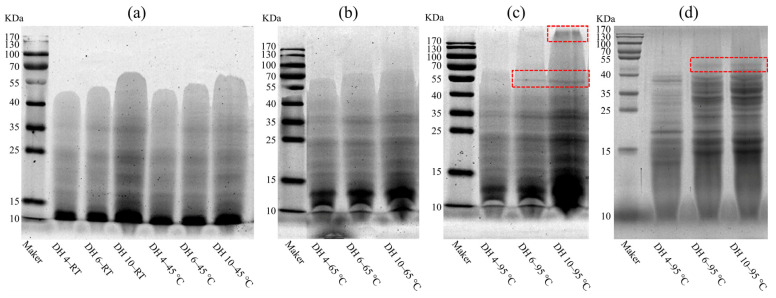
Electropherograms of GPs heated at 45 (**a**), 65 °C (**b**), 95 °C (**c**), and of reduced GPs (**d**). DH 4, DH 6, and DH 10 represent the degree of hydrolysis 4.0%, degree of hydrolysis 6.0%, and degree of hydrolysis 10.0%, respectively. The red boxes denote the different parts when heated at 95 °C.

To investigate the effect of thermal treatment on GPs and to further assess the mechanism of the DH on the formation of AFs, the molecular weight distribution of GPs after 120 min of heating was measured using SDS-PAGE, as shown in Figure 2 and Figure S2. At RT, the range of bands on the spectrograms of GPs with a different DH was significantly different (Figure 2a), with the band range increasing with the increasing DH. The bands’ range of DH 4, DH 5, and DH 10 were 10–55 KDa, 10–55 KDa, and 10–70 KDa, respectively. Moreover, the intensity of the bands also increased with the increase of the DH. Especially in DH 10–RT, which exhibited the greatest intensity and range of bands. This indicated that the greater the DH, the wider the range of molecular weights and the greater the number of polypeptides contained in the GP solution. The bands’ range and intensity of GPs heated at 45 °C for 120 min did not change significantly, compared to GPs left at RT for 120 min, indicating that breading hydrogen bonds was not adequate to drive the polymerization between polypeptides.

As shown in Figure 2b, heating at 65 °C increased the range of bands, with DH 4, DH 5, and DH 10 expanding to 10–70 KDa, 10–70 KDa, and 10–100 KDa, respectively. This phenomenon was more pronounced after heating at 95 °C (Figure 2c). Hydrophobic interactions became stronger with the increasing heating temperature, which induced the non-covalent aggregation between wheat proteins [21,28]. These changes at the molecular scale could be regarded here as rearrangements of the GPs through hydrophobic interactions, resulting in the formation of a polymer with a larger molecular weight. In addition, a new band with a molecular weight of approximately 55 KDa was observed (highlight region) at DH 6 and DH 10, when heating at 95 °C. The DH 10 GPs formed a macropolymer (KDa > 170, highlight region) after heating at 95 °C. When temperatures reached 90 °C, the covalent polymerization of proteins was triggered via the SH-SS exchange reactions [18]. The disappearance of the macropolymer region after reduction (Figure 2d) indicated that the aggregation of the macropolymer region was maintained by the cross-linking of the disulfide bonds. In contrast, the persistence of the new band at approximately 55 KDa proved that the formation of this band was not formed by the cross-linking of the disulfide bonds, but was driven by enhanced hydrophobic interactions. Combined with the result of Section 3.1, the fluorescence intensity of DH 6 and DH 10 GPs increased rapidly after heating at 95 °C for 90–120 min, suggesting that the appearance of new bands at approximately 55 KDa was associated with the formation of AFs and also indicated that hydrophobic interactions were relevant for the fibrillation of the GPs.

### 2.4. Molecular Constitution of GPs after Thermal Treatment

SE-HPLC is a chromatographic technique that uses elution times and peak areas to qualitatively and quantitatively analyze the molecular composition of a sample, based on the size of the molecules [29]. The chromatograms of the GPs under different thermal treatments are shown in Figure 2. The peptides were roughly divided into two parts depending on the elution time, with those with an elution time between 17.5 and 21.5 min being referred to as high molecular weight peptides, and those with an elution time between 21.5 and 25 min being referred to as low molecular weight peptides.

**Figure 3:**
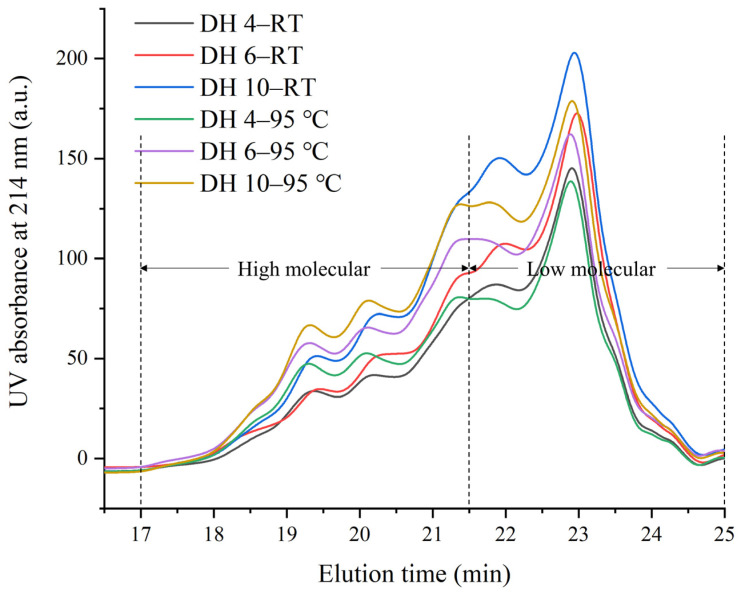
SE-HPLC profiles of heated and unheated gluten polypeptides. DH 4, DH 6, and DH 10 represent the degree of hydrolysis 4.0%, the degree of hydrolysis 6.0%, and the degree of hydrolysis 10.0%, respectively.

At RT, the peak emergence time of the GP curves was not significantly different for the different DHs, but the peak heights were quite different and increased significantly with the increasing DH. This indicated that the molecular composition of the different DH GPs hydrolyzed with trypsin was not distinctly different but that the content of individual constituent molecules increased significantly with the increasing DH. The content of GPs of different molecular weights changed after heating at 95 °C. A comparison of the DH 4–RT and DH 4–95 °C curves showed that the content of the low molecular weight peptides decreased and the content of the high molecular weight peptides increased after heating. This phenomenon was observed, not only in DH 4, but also in DH 6 and DH 10 after heating, and the trend became more pronounced with the higher DH. AFs had a very stable structure and free energy, ensuring that they were not damaged by external physico-chemical reactions [30]. While most protein fractions were broken down in 2% SDS, AFs were resistant to structural damage by SDS [30]. This might imply that the high content of high molecular weight peptides could be due to the aggregation of low molecule weight peptides to form AFs. The failure to observe the formation of the macropolymer from DH 10 GPs (DH 10–95 °C, Figure 3) by chromatogram was potentially due to its excessive molecular weight (KDa > 170) failing to pass through the filter membrane.

### 2.5. Morphology of GPs after Thermal Treatment

**Figure 4:**
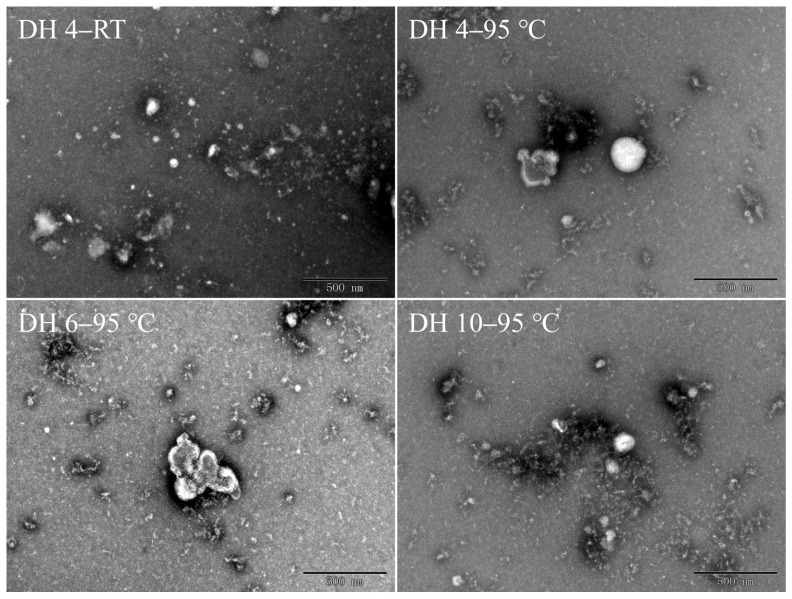
Transmission electron microscopy images of gluten polypeptides after heating for 120 min. DH 4, DH 6, and DH 10 represent the degree of hydrolysis 4.0%, degree of hydrolysis 6.0%, and degree of hydrolysis 10.0%, respectively.

The morphology of the GPs was studied by TEM, as shown in Figure 4. The DH 4 GPs at RT exhibited a scattered and disordered distribution, with peptide molecules of different sizes distributed throughout the visual field (DH 4–RT, Figure 4). Here, the peptide monomers were mostly ovoid, due to intramolecular hydrogen bonding and external hydrophobic interactions, with no obvious aggregation behavior between monomers existing independently. Following the heating at 95 °C for 120 min, DH 4 GPs lost their ovoid structure and the peptide monomers were fibrillated, with the monomers remaining separately dispersed in the field of view. In contrast to DH 4 GPs, the monomers of DH 6 and DH 10 GPs formed dense worm-like structures by clustering with each other, and the tendency for such clustering increases with the increasing DH. This indicated that the smaller molecular weight was more favorable for fibrillation. In addition, Lambrecht et al. found that trypsinized wheat proteins formed long and straight fibrils after 40 h of heating at 85 °C [23]. Ji et al. found that soy protein isolates heated at 95 °C for 1 h formed short and dispersed fibers, but after 4 h, the fibers were long and straight [31]. This suggested that worm-like oligomers were precursors to the formation of long fibers.

## 3. Materials and Methods

### 3.1. Materials

Wheat flour (68.94% carbohydrate, 14.71% moisture, 12.85% protein, 2.84% fat, and 0.66% ash) was purchased from COFCO Flour Industry Co., Ltd. (Zhengzhou, China). All chemical reagents in the experiments were of analytical grade.

### 3.2. Preparation of the Wheat Gluten and Wheat Gluten Polypeptides (GPs)

Gluten fractions were obtained from wheat flour, according to the description in the approved method 38-10.01 (AACCI, 2000), then lyophilized (−50 °C, 0.040 mbar, for 24 h) in an ALPHA 1–2 LD plus lyophilizer (Marin Christ, Osterode, Germany) and powdered (passed 100 mesh sieve).

The hydrolysis of the wheat gluten was carried out according to the method described by Lambrecht et al. with some modifications [23]. First, 15 g of wheat gluten was dispersed in 300 mL of deionized water and heated in a water bath at 40 °C, and the pH was adjusted to 8.0 using 1 M sodium hydroxide. Then, it was hydrolyzed with trypsin (2500 U/mg, T819002, Macklin, Shanghai, China) to a degree of hydrolysis (DH) of 4.0% (DH 4), 6.0% (DH 6), and 10.0% (DH 10). The trypsin to substrate protein ratio for DH 4, DH 6, and DH 10 were 1:150, 1:20, and 1:10, respectively. During the reaction, 1 M sodium hydroxide was added continuously to maintain the pH at 7.0. Upon reaching the appropriate DH, the enzyme was inactivated in a water bath at 95 °C for 5 min. Next, the samples were cooled to room temperature in an ice bath, centrifuged at 18,000× *g* for 15 min, and the supernatant collected. The supernatant was adjusted to pH 7.0 with 0.05 M HCl, lyophilized, and configured to a concentration of 2.0% (*w*/*v*), and used for the subsequent experiments.

DH was defined as the number of peptide bonds hydrolyzed (h) as a percentage of a total number of peptide bonds present in a unit weight of wheat gluten (h_tot_, 8.3 mequiv/g protein) and was calculated as follows:

DH (%) = h/h_tot_ = B × M_b_ × 100/α × m_p_ × h_tot_

where B is the total amount of sodium hydroxide added, M_b_ is the molar concentration of sodium hydroxide added, α is the degree of dissociation of the α-NH_3_^+^ (0.834 at 40 °C), and m_p_ is the mass of protein.

### 3.3. Self-Assembly of Wheat Peptides

The samples obtained by Section 2.2 were heated at 45 °C (reduced hydrogen bonding interactions), 65 °C (enhanced hydrophobic interactions), and 95 °C (facilitated disulfide bond cross-linking) for 30 min, 60 min, 90 min, and 120 min.

### 3.4. Thioflavin T Fluorescence Measurements

Thioflavin T (ThT) fluorescence was measured according to the method described by Ji et al. with some modifications [31]. Briefly, 1.90 mL of the sample was mixed with 100 μL of 200 μM ThT in a dark room for 1 h. Next, the sample was detected using an F-7100 fluorescence spectrophotometer (Hitachi, Tokyo, Japan) with excitation wavelengths at 440 nm and emission wavelengths from 460 to 600 nm.

### 3.5. Congo Red Spectral Shift Analysis

The Congo red spectrum was measured, according to the method described by Hudson et al. [20]. Briefly, a 9.0 mL sample was incubated with 1 mL of 0.01% (*w*/*v*) Congo red solution for 30 min in a dark room. The shift in absorbance from 460 to 540 nm was measured using a UV-Vis spectrophotometer (UV1901, Aoxi, Shanghai, China) with a resolution of 5 nm.

### 3.6. Fourier Transform Infrared (FT-IR) Spectroscopy

The obtained samples were lyophilized (−50 °C, 0.040 mbar, 24 h) using an ALPHA 1–2 LD plus lyophilizer (Marin Christ, Osterode, Germany) and powdered (passed 100 mesh sieve). Next, the lyophilized powder was mixed with KBr at a ratio of 1:100. About 1 g of the mixed sample was pressed into a transparent sheet and then placed in an FT-IR spectrometer (Thermo Scientific, Waltham, MA, USA), where the sample was scanned 16 times at a resolution of 4 cm^−1^ over a scan range of 400–4000 cm^−1^. Next, the amide I region (1600–1700 cm^−1^) was peaked and analyzed using Peak Fit v4.12 software (Systat Software Inc., San Jose, CA, USA), as described by Wang et al. [32].

### 3.7. Sodium Dodecyl Sulfate Polyacrylamide Gel Electrophoresis (SDS-PAGE)

SDS-PAGE was performed with a 12% separation gel (pH 8.8) and a 5% concentration gel (pH 6.8), according to the method of the previous study [14]. Briefly, 0.5 mL samples were mixed with 1 mL of loading buffer (62.5 mM Tris-HCL, pH 6.8, containing 2% (*w*/*v*) SDS, 10% (*v*/*v*) glycerol, and 0.1% (*w*/*v*) bromophenol blue), shaken at 200 rpm for 2 h at 37 °C. The reducing buffer was added to the original loading buffer with 5% (*v*/*v*) β-mercaptoethanol. Then centrifuged at 12,000× *g* for 15 min, the supernatant was collected and boiled for 5 min and cooled to room temperature. A 10 μL supernatant was loaded into each well and run at 120 V.

### 3.8. Size-Exclusion High-Performance Liquid Chromatography (SE-HPLC)

The lyophilized sample (1.0 mg) was dissolved in 1 mL of 0.05 M sodium phosphate buffer (pH 6.8, containing 2.0% (*w*/*v*) SDS) and shaken for 60 min (25 °C). The samples were centrifuged at 10,000× *g* for 10 min. The supernatant was filtered through a 0.45 μm polyethersulfone microporous filter and uploaded onto a Biosep-SEC-S4000 column (Phenomenex, Torrance, CA, USA). The elution solution was a 50% (*v*/*v*) mixture of acetonitrile and water containing 0.1% trifluoroacetic acid at an elution rate of 0.5 mL/min. The detection was performed using a 1260 Infinity HPLC (Agilent, Santa Clara, CA, USA) automated injection detection system at UV 214 nm.

### 3.9. Transmission Electron Microscopy (TEM)

The microscopic morphology of the samples was observed using a JEM 1200EX TEM (JEOL, Tokyo, Japan). Then, a 2 μL aliquot sample was transferred to the surface of a carbon-coated 400 mesh nickel grid. The grid surface was washed three times with 10 μL of ultrapure water and negatively stained with 10 μL of 2% (*w*/*v*) uranyl acetate solution. The excess solution was blotted off with filter paper. The grids were allowed to dry, and the results were observed at a magnification of 25,000–64,000 with an excitation voltage of 80 kV.

### 3.10. Statistical Analysis

All data were expressed as mean ± standard deviation (SD) of at least three independent experiments. The data were analyzed to compare the means using SPSS version 13.0 (SPSS Inc., Chicago, IL, USA). The statistical analysis was performed using Tukey’s post hoc test, with *p* < 0.05 indicating a significant difference.

## 4. Conclusions

In this study, the thermal effects and DH on the formation of AFs via the self-assembly of GPs was investigated. The results from the ThT fluorescence intensity showed that the strengthening of the hydrophobic interactions contributed significantly to the fibrillation of GPs. The DH 10 GPs showed the most vigorous fluorescence intensity (5552 a.u.) after heating at 95 °C for 120 min, which might be due to the higher DH exposing more hydrophobic groups and being more susceptible to hydrophobic interactions. The results of FT-IR spectroscopy indicated that enhanced hydrophobic interactions facilitated the conversion of the random coil and α-helix to the β-sheet. The molecular weight of GPs increased upon heating, which meant that more structurally stable AFs were formed. In addition, the morphology presented by TEM showed that the DH 10 GPs had the most significant degree of worm-like aggregation, which was also related to their susceptibility to hydrophobic interactions. This paper provides an initial exploration of the possibilities and mechanisms of nanomaterials using natural plant-based proteins. It was found that hydrophobic interactions may be the primary driver affecting the occurrence of the amyloid aggregation in GPs. However, how gluten is induced to form AFs with long and straight three-dimensional structures remains unknown and will be the focus of further research.

## Figures and Tables

**Table 1:** Effect of the DH and thermal treatment on the secondary structure of GPs heated for 120 min.

Sample	β-Sheet (Fibrillation)/%	β-Sheet (Native)/%	Random Coil/%	α-Helix/%	β-Turn, Antiparallel β-Sheet/%
DH 4–RT	4.98 ± 0.22 ^a^	7.63 ± 0.36 ^a^	22.09 ± 0.80 ^f^	30.98 ± 0.76 ^d^	35.16 ± 0.88 ^a^
45 °C	12.19 ± 0.22 ^b^	-	19.12 ± 0.73 ^bcde^	29.28 ± 0.58 ^cd^	39.41 ± 0.07 ^b^
65 °C	12.94 ± 0.37 ^bc^	-	18.96 ± 0.82 ^bcde^	26.76 ± 1.25 ^b^	41.33 ± 2.44 ^b^
95 °C	13.61 ± 0.11 ^cd^	-	18.46 ± 0.06 ^bcd^	26.42 ± 0.14 ^b^	41.51 ± 0.19 ^b^
DH 6–RT	5.08 ± 0.53 ^a^	8.18 ± 0.72 ^a^	20.08 ± 0.82 ^de^	30.42 ± 0.48 ^d^	36.24 ± 0.75 ^a^
45 °C	13.23 ± 0.00 ^bcd^	-	18.46 ± 0.10 ^bcd^	27.18 ± 0.11 ^bc^	41.12 ± 0.21 ^b^
65 °C	14.22 ± 0.67 ^cd^	-	17.67 ± 0.15 ^bc^	27.40 ± 1.80 ^bc^	40.70 ± 0.98 ^b^
95 °C	17.81 ± 0.90 ^e^	-	17.40 ± 0.30 ^b^	25.38 ± 0.60 ^b^	39.41 ± 0.01 ^b^
DH 10–RT	4.60 ± 0.42 ^a^	8.23 ± 0.23 ^a^	20.43 ± 0.96 ^e^	30.14 ± 1.17 ^d^	35.76 ± 0.83 ^a^
45 °C	13.42 ± 0.00 ^bcd^	-	19.28 ± 0.49 ^de^	27.50 ± 0.14 ^bc^	39.80 ± 0.63 ^b^
65 °C	14.55 ± 0.53 ^d^	-	17.52 ± 0.65 ^bc^	26.67 ± 0.30 ^b^	40.26 ± 1.60 ^b^
95 °C	21.42 ± 0.96 ^f^	-	15.55 ± 0.52 ^a^	23.21 ± 0.34 ^a^	39.81 ± 0.78 ^b^

Means with small superscript letters within the same column are significantly different at *p* < 0.05.

## Data Availability

The data used to support the findings of this study are available from the corresponding author upon request.

## Supplementary Materials

The following supporting information can be downloaded at: https://www.mdpi.com/article/10.3390/molecules28020834/s1, Figure S1: FT-IR spectra of DH 4 (a), DH 6 (b), and DH 10 (c) under different heating temperatures. DH 4, DH 6, and DH 10 represent the degree of hydrolysis 4.0%, degree of hydrolysis 6.0%, and degree of hydrolysis 10.0%, respectively; Figure S2: Original electropherograms of GPs heated at 45 (a), 65 °C (b), 95 °C (c), and of reduced GPs (d). DH 4, DH 6, and DH 10 represent the degree of hydrolysis 4.0%, degree of hydrolysis 6.0%, and degree of hydrolysis 10.0%, respectively.

## Sample Availability

Samples of the compounds GPs, wheat gluten, and wheat flour are available from the authors.
