# Supplementary material for: Effect of Thermal Treatment on the Self-Assembly of Wheat Gluten Polypeptide
Source: Molecules. 2023 Jan 14;28(2):834. doi: 10.3390/molecules28020834 (PMC9862778; doi:10.3390/molecules28020834)
Supplement: Supplementary file 1 [file molecules-28-00834-s001.zip › molecules-2097121-supplementary.pdf]

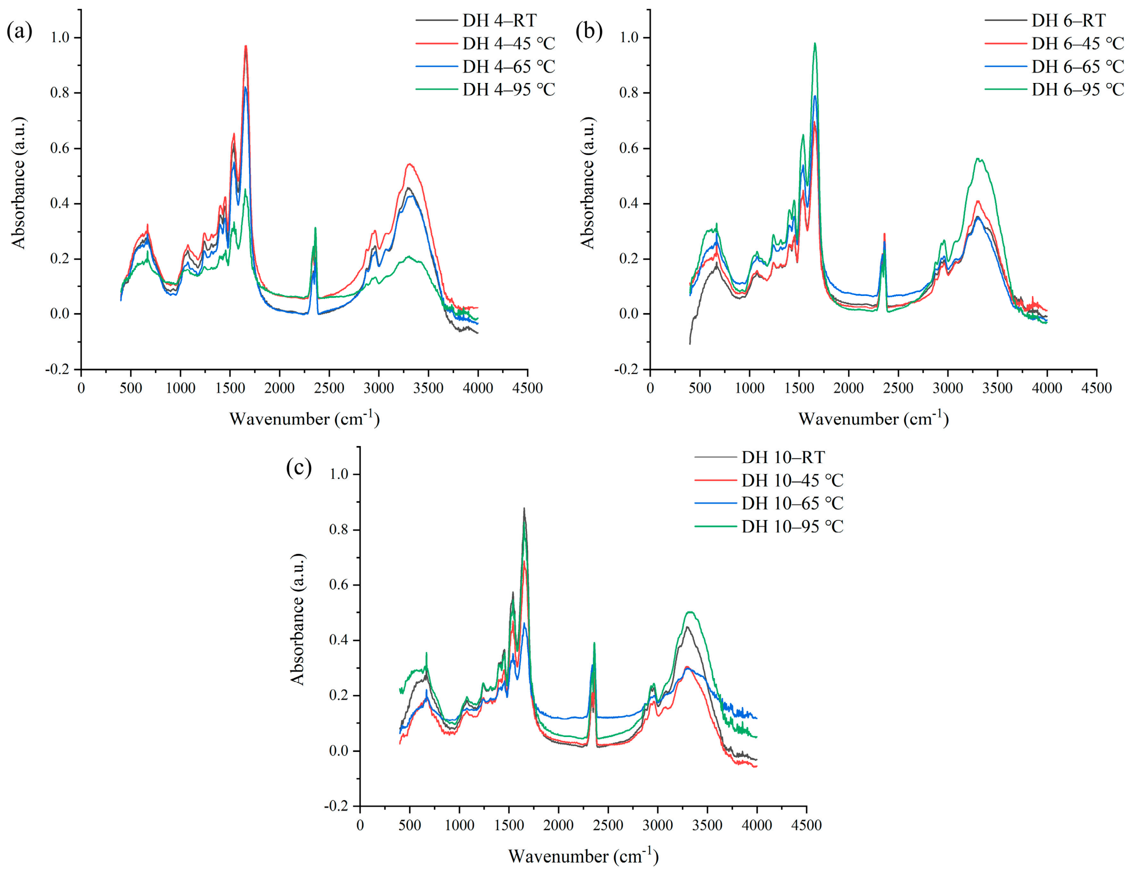

Figure S1. FT-IR spectra of DH 4 (**a**), DH 6 (**b**), and DH 10 (**c**) under different heating temperatures. DH 4, DH 6, and DH 10 represent the degree of hydrolysis 4.0%, degree of hydrolysis 6.0%, and degree of hydrolysis 10.0%, respectively

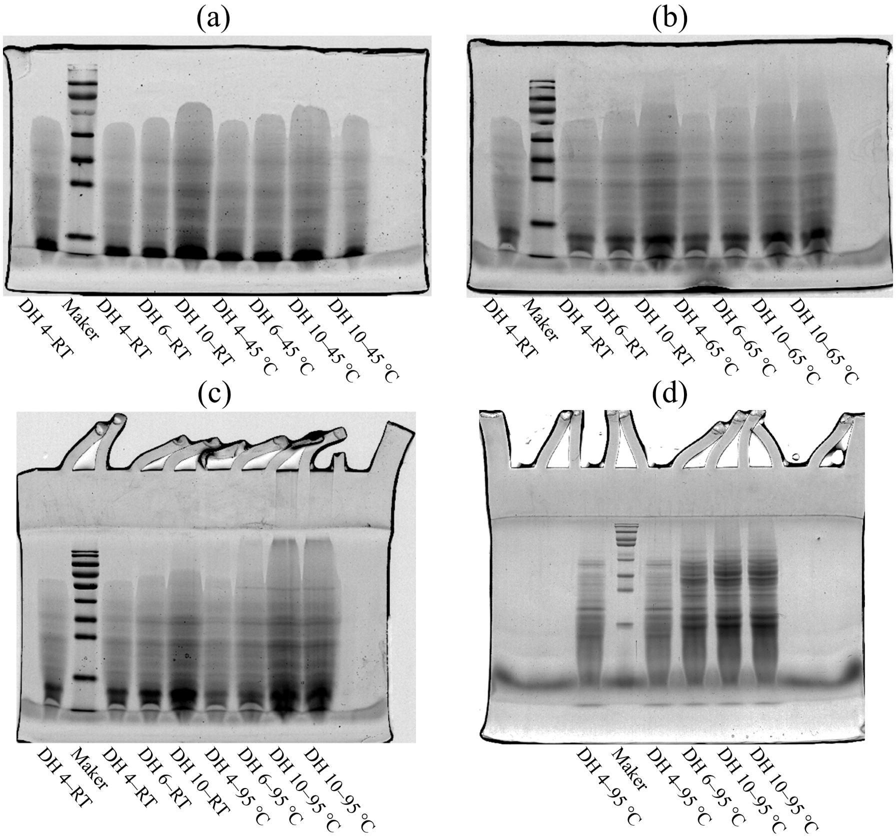

Figure S2. Original electropherograms of GPs heated at 45 (**a**), 65 °C (**b**), 95 °C (**c**), and of reduced GPs (**d**). DH 4, DH 6, and DH 10 represent the degree of hydrolysis 4.0%, degree of hydrolysis 6.0%, and degree of hydrolysis 10.0%, respectively.
